# Integrative and Conjugative Elements-Positive *Vibrio parahaemolyticus* Isolated From Aquaculture Shrimp in Jiangsu, China

**DOI:** 10.3389/fmicb.2019.01574

**Published:** 2019-07-18

**Authors:** Yu He, Shuai Wang, Jianping Zhang, Xueyang Zhang, Fengjiao Sun, Bin He, Xiao Liu

**Affiliations:** ^1^College of Food Biological Engineering, Xuzhou University of Technology, Xuzhou, China; ^2^Key Construction Laboratory of Food Resources Development and the Quality Safety in Jiangsu, Xuzhou University of Technology, Xuzhou, China; ^3^College of Environmental Engineering, Xuzhou University of Technology, Xuzhou, China; ^4^Logistics & Security Department, Shanghai Civil Aviation College, Shanghai, China; ^5^Environment Monitoring Station, Zaozhuang Municipal Bureau of Ecology and Environment, Zaozhuang, China; ^6^Henan Key Laboratory of Cold Chain Food Quality and Safety Control, Zhengzhou University of Light Industry, Zhengzhou, China

**Keywords:** *Vibrio parahaemolyticus*, antimicrobial susceptibility, integrative and conjugative elements, heavy metal resistance, genotypes

## Abstract

The development of multidrug- and toxin-resistant bacteria as a result of increasing industrialization and sustained and intense antimicrobial use in aquaculture results in human health problems through increased incidence of food-borne illnesses. Integrative and conjugative elements (ICEs) are self-transmissible mobile genetic elements that allow bacteria to acquire complex new traits through horizontal gene transfer and encode a wide variety of genetic information, including resistance to antibiotics and heavy metals; however, there is a lack of studies of ICEs of environmental origin in Asia. Here, we determined the prevalence, genotypes, heavy metal resistance and antimicrobial susceptibility of 997 presumptive strains of *Vibrio parahaemolyticus* (*tlh*^+^, *tdh*^–^), a Gram-negative bacterium that causes gastrointestinal illness in humans, isolated from four species of aquaculture shrimp in Jiangsu, China. We found that 59 of the 997 isolates (5.9%) were ICE-positive, and of these, 9 isolates tested positive for all resistance genes. BLAST analysis showed that similarity for the eight strains to *V. parahaemolyticus* was 99%. Tracing the *V. parahaemolyticus* genotypes, showed no significant relevance of genotype among the antimicrobial resistance strains bearing the ICEs or not. Thus, in aquaculture, ICEs are not the major transmission mediators of resistance to antibiotics or heavy metals. We suggest future research to elucidate mechanisms that drive transmission of resistance determinants in *V. parahaemolyticus*.

## Introduction

Microbes rapidly acquire or donate new genes and phenotypes through the process of horizontal gene transfer between organisms that is a key driver of microbial evolution (Pan et al., 2019). Integrative and conjugative elements (ICEs) are self-transmissible modular mobile genetic elements (MGEs) integrated into a host genome that are passively propagated during chromosomal replication and cell division, and mediate the acquisition of complex new traits in bacteria (Johnson and Grossman, 2015). Recent studies have shown these MGEs contain cargo genes encoding traits including resistance, virulence, novel carbon source metabolism, and degradation of aromatic compounds, that may benefit the recipient bacteria (Rubio-Cosials et al., 2018; Xu et al., 2018). ICEs tend to be mosaic and modular, ranging from 20 to >500 kb. ICEs are excised from the host chromosome and then transfer to recipients via conserved conjugation machinery in the type IV secretion system (Flores-Ríos et al., 2019), prior to reintegration into the host chromosome.

*Vibrio parahaemolyticus* is a Gram-negative, halophilic, mesophilic, aerobic bacterium common in warm climate marine and estuarine environments. Pathogenic strains in food cause serious health issues to humans, including gastroenteritis, septicemia, and wound infection (He et al., 2016). Shrimp represents an important reservoir of *V. parahaemolyticus*, particularly in fresh and refrigerated stock, but it is also recorded from frozen stock. China is the world’s largest producer of aquatic products (He et al., 2016). However, industrial development and use of antimicrobials in aquaculture have led to increased heavy metal pollution and development of multidrug resistant (MDR) bacteria that are problematic in many aquatic systems as they drive incidence of food-borne illnesses (Lopatek et al., 2018). Previous studies have revealed that bacteria can acquire resistance via conjugation or transformation to induce a wide variety of disease and adapt to the harsh environment (Matyar, 2012). The World Health Organization (WHO) produced a global map of antimicrobial resistance, warning that a “post-antibiotic” world could soon become a reality in April 2014 (WHO, 2014). Recent studies indicated that drugs which were once lifesavers are now worthless, for instance, chloramphenicol, once a physician’s first choice against typhoid, is no longer effective in many parts of the world and resistance has spread around the world (Woolhouse and Farrar, 2014). Antimicrobial resistance is a global problem that requires global solutions, better surveillance is essential. Nevertheless, to date, no global approaches were conducted on further demonstrating the characterization of the *V. parahaemolyticus* isolates present in shrimp-production industry, despite their great significance in economy and human health.

The discovery and early studies of ICEs were stimulated by interest in bacterial resistance to antibiotics and heavy metals, and how that resistance was spread. MGEs with ICE-like properties have been described in several species of Gammaproteobacteria, particularly *Vibrio* (Liu et al., 2019). However, few studies report on ICEs in *V. parahaemolyticus* isolates from Asia. Hence, in this study, we focused on analyzing the *V. parahaemolyticus* strains from different shrimp samples in Jiangsu, China to determine the antibiotic and heavy metal resistance of these bacteria and to investigate the relationship between antibiotic and heavy metal. Molecular characterization and phenotypes of antibiotic resistance and heavy metals have been characterized. The information will facilitate the better understanding of this bacterium and facilitate related risk assessment and health management for consuming seafood.

## Materials and Methods

### Sample Collection

Freshwater shrimp (*Procambarus clarkii*, *Macrobrachium nipponense*, *Penaeus vannamei*, and *Macrobrachium rosenbergii*), which are commonly shrimp breeds in China were collected once a month from Xuzhou Kaiming Fish Market, Jiangsu, China from May to October 2016–2018. *P. clarkii* and *M. nipponense* are key economic species in Jiangsu; *P. vannamei* and *M. rosenbergii* are cultured widely in the littoral provinces of southeastern China. We randomly collected samples of each species following a modified version of a standard protocol (Kaysner and DePaola, 2004). Samples were placed in sterile plastic bags (Shanghai Sangon Biological Engineering Technology and Services Co., Ltd., Shanghai, China) and taken to the laboratory on ice for homogenization within 2 h of collection.

### *V. parahaemolyticus* Isolation and Identification

*Vibrio parahaemolyticus* was isolated and identified as described by the Chinese Government Standard (GB17378-2007) and He et al. (2016). Shrimp (25 g) were rinsed with sterile saline solution then homogenized for 60 s in sterile bags (Stomacher) containing 225 mL of sterile saline. Serial 10-fold dilutions (to 1:10^4^) were prepared, and 100 μl of each dilution were spread on thiosulfate citrate-bile salts-sucrose plates (TCBS; Beijing Land Bridge Technology Company Ltd., Beijing, China) which were incubated for 16 h at 37°C. Putative *V. parahaemolyticus* colonies (which are green on TCBS) were placed separately in wells of 96-well microtiter plates containing 200 μl of sterile alkaline peptone water plus 3% NaCl (pH 8.5 ± 0.2).

### Screening and Identification of Virulence and ICE Genes

Presumptive *V. parahaemolyticus* colonies (*N* = 25 per species of shrimp) were selected, screened, and identified using PCR-based screening of the species-specific marker gene thermolabile hemolysin (*tlh*). Table 1 lists primers used in this study. In *tlh*-positive *V. parahaemolyticus* strains, the virulence-associated genes thermostable direct hemolysin (*tdh*) and TDH-related hemolysin (*trh*), SXT/R391-like ICE conserved genes (*int*, *attR*, *traC*, *setR*, and *traI*), and the typical resistance genes for streptomycin (*strA*/*strB*), trimethoprim (*dfrA1*/*dfr18*), and sulfamethoxazole/trimethoprim (*sul2*) were identified using primers as described by He et al. (2015a, 2016) and Beker et al. (2018). Strain taxonomy was determined from 16S rRNA gene sequences, obtained using primer pair 27F and 1492R; sequencing was performed by Shanghai Sangon Biological Engineering Technology and Services Co., Ltd., (Shanghai, China) (He et al., 2016). *V. parahaemolyticus* ATCC33846 (*tdh*^+^, *trh*^–^) and ATCC17802 (*tdh*^–^, *trh*^+^) were used as positive controls. Genomic DNA was prepared using a MiniBest bacterial genomic DNA extraction kit (v. 2.0; Japan TaKaRa BIO, Dalian, China). DNA was amplified using a Mastercycler pro PCR thermal cycler (Eppendorf, Hamburg, Germany). DNA sequences were assembled into contigs using ContigExpress software^1^. Protein functions were analyzed using BLAST^2^.

**TABLE 1:** Primers used in this study.

**Primer**	**Nucleotide sequence (5′–3′)**	**Target genes**	**References**
27F	AGAGTTTG ATCCTGGCTCAG	16S rDNA	Weisburg et al.,1991
1492R	GGTTACC TTTTACGACTTG		
L-TLH	AAAGCGGAT TATGCAGAAGCACTG	*tlh*	Panicker et al.,2004
R-TLH	ACTTTCTAGC ATTTTCTCTGC		
Tdh-1	CCATCTGTCC CTTTTCCTGCC	*tdh*	Panicker et al.,2004
tdh-4c	CCACTACCA CTCTCATATGC		
VPTRH-L	TTGGCTTCG ATATTTTCAGTATCT	*trh*	Panicker et al.,2004
VPTRH-R	CATAACAAAC ATATGCCCATTTCCG		
traI-F traI-R	GCAAGT CCTGATCCGCTATC	*traI*	Bani et al.,2007
	CCAGGGCAT CTCATATGCGT		
traC-F	TTGACGCT GTTTTCACCAACG	*traC*	Bani et al.,2007
traC-R	GGCACGAC CTTTTTTCTCCC		
setR-F	ACGGCGGA GATGTTTTTGT	*setR*	Bani et al.,2007
setR-R	GTGCGCC AATGCTCAGTT		
attR-F	GGTTTAG CCACAGTTGTTC	*attR*	Song et al.,2013
attR-R	CGTCAG GGTGCGAGAT		
Int-F	GACGCATT TCATCCAGG	*int*	Song et al.,2013
Int-R	GCAACAG CGGGTAGACA		
SUL2-F	AGGGGGC AGATGTGATCGC	*sul2*	Falbo et al.,1999
SUL2-B	TGTGCGG ATGAAGTCAGCTCC		
STRA-F	TTGATGT GGTGTCCCGCAATGC	*strA*	Dalsgaard et al.,1999
STRA-B	CCAATCG CAGATAGAAGGCAA		
strB-F	GGCACCC ATAAGCGTACGCC	*strB*	Dalsgaard et al.,1999
strB-R	TGCCGAG CACGGCGACTACC		
dfr1-F	CGAAGAA TGGAGTTATCGGG	*dfrA1*	Iwanaga et al.,2004
dfr1-B	TGCTGG GGATTTCAGGAAAG		
TMP-F	TGGGTAA GACACTCGTCATGGG	*dfr18*	Falbo et al.,1999
TMP-B	ACTGCCG TTTTCGATAATGTGG		

### Susceptibility to Antimicrobials and Heavy Metals

*In vitro* susceptibility of isolates to antimicrobial agents according to the guidance of the Performance Standards for Antimicrobial Disk Susceptibility Tests of the Clinical and Laboratory Standards Institute (CLSI) (2006, Approved Standard-Ninth Edition, M2-A9, Vol. 26 No. 1) following the approach described by Song et al. (2013) and He et al. (2015a). Mueller-Hinton agar medium (Oxoid, United Kingdom), and the discs (Oxoid, United Kingdom) were used in this study. Examined antimicrobial agents included: 30 μg of chloramphenicol (CHL); 10 μg of gentamicin (CN); 25 μg of sulfamethoxazole/trimethoprim 19:1 (SXT); 5 μg of rifampin (RIF); 30 μg of tetracycline (TET); 10 μg of ampicillin (AMP); 100 μg of spectinomycin (SPT); 30 μg of kanamycin (KAN); 5 μg of trimethoprim (TM); and 10 μg of streptomycin (STR). The assays were performed in triplicate experiments, and reference strain *Escherichia coli* ATCC25922 was purchased from the Institute of Industrial Microbiology (Shanghai, China) and used for quality control. Broth Dilution Testing (microdilution) was used to measure quantitatively the minimal inhibitory concentration (MIC) *in vitro* of the tested heavy metals against the strains, according to the Methods for Dilution Antimicrobial Susceptibility Tests for Bacteria That Grow Aerobically (2006, CLSI, Approved Standard-Seventh Edition, M7-A7, Vol. 26 No. 2). The heavy metals tested were: NiCl_2_, CrCl_3_, CdCl_2_, PbCl_2_, CuCl_2_, ZnCl_2_, BaCl_2_, and HgCl_2_ (Sinopharm Chemical Reagent Co., Ltd., Shanghai, China); *E. coli* K12 MG1655 strain was used as the control.

### Molecular Typing of *V. parahaemolyticus* Strains

*Vibrio parahaemolyticus* was cultured in Luria-Bertani broth (Beijing Land Bridge Technology Co.) following methods described by He et al. (2015b). Genomic DNA was extracted using a CHEF Bacterial DNA Plug Kit (Bio-Rad Laboratories, Hercules, CA, United States). Agarose plugs were prepared by mixing equal volumes of cell suspension, and each plug was digested using *Not*I (Japan TaKaRa BIO). Electrophoresis was performed at 6 V/cm, 14°C, at a field angle of 120°, using 1% SeaKem Gold agarose (Lonza, Basel, Switzerland). Pulsed-field gel electrophoresis (PFGE) patterns were visualized under 260-nm light; images were recorded using the UVP EC3 Imaging system (UVP LLC), and data were analyzed using NTSYSpc 2.10e software.

## Results and Discussion

### *V. parahaemolyticus* Isolation and Identification

**FIGURE 1:**
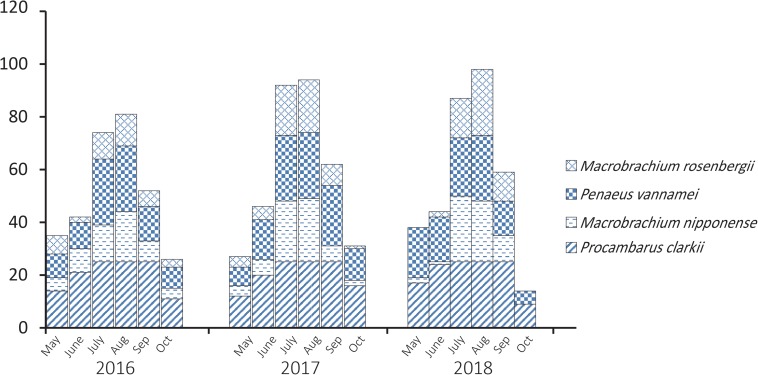
Temporal patterns of prevalence of *Vibrio parahaemolyticus* isolated from aquaculture shrimp.

*Procambarus clarkii*, *M. nipponense*, *P. vannamei*, and *M. rosenbergii* are common species of shrimp consumed in Jiangsu, China, and PCR analysis showed that 997 of 1800 (55.4%) bacterial isolates obtained from them tested positive for the *V. parahaemolyticus*-specific *tlh* gene. There were distinct temporal patterns of *tlh*-positive prevalence (Figure 1); over the 3-year study, *tlh* gene abundance was greater during the summer months (July and August) when temperature is highest.

### Prevalence of Virulence Associated-Genes and Conserved ICE Genes

Pathogenic *V. parahaemolyticus* produces two major toxic proteins, TDH and TRH, that are important in the diarrheal diseases caused by this species (Boyd et al., 2008). We tested the 997 *tlh*-positive strains for the presence of virulence-associated toxin genes *tdh* and *trh*, and found that most isolates were considered not virulent; we did not amplify *tdh* from any isolate, and amplified *trh* from only two isolates (obtained from *P. clarkii* in August 2018). Similar very low incidence of pathogenic *V. parahaemolyticus* has been reported in many non-clinical samples (He et al., 2016; Hu and Chen, 2016; Martinez-Urtaza et al., 2016; Lopatek et al., 2018; Zhao et al., 2018; Jiang et al., 2019).

Analysis of the highly conserved core genes of SXT/R391-like ICEs revealed that 59 (5.9%) of the isolates tested positive for all five genes (*int*, *attR*, *traC*, *setR*, and *traI*). Occurrence was highest in isolates recovered from *P. clarkii* (33.9%), followed by *P. vannamei* (32.2%), *M. nipponense* (20.3%), and *M. rosenbergii* (13.6%), and resistance genes *strA*/*strB*, *dfrA1*/*dfr18*, and *sul2* were recorded in 25.4, 15.3, and 33.9% of the 59 isolates, respectively, with nine strains testing positive for all three resistance genes (Table 2).

**TABLE 2:** Genotypes of *V. parahaemolyticus* isolated from shrimp.

**Source (species)**	**Strain no.**	**Sample date**	***tlh***	**Virulence-associated genes**		**ICEs conserved-core genes**					**Resistance genes**		
				***tdh***	***trh***	***int***	***attR***	***traI***	***traC***	***setR***	***strA*/*strB***	***dfrA1*/*dfr18***	***sul2***
*Procambarus clarkii*	VpJHY1	July 2016	+	−	−	+	+	+	+	+	−	−	+
	VpJHY2	July 2016	+	−	−	+	+	+	+	+	−	−	−
	VpJHY3	August 2016	+	−	−	+	+	+	+	+	+	−	+
	VpJHY4	August 2016	+	−	−	+	+	+	+	+	+	+	+
	VpJHY5	August 2016	+	−	−	+	+	+	+	+	−	−	−
	VpJHY6	July 2017	+	−	−	+	+	+	+	+	−	−	−
	VpJHY7	August 2017	+	−	+	+	+	+	+	+	+	−	+
	VpJHY8	August 2017	+	−	−	+	+	+	+	+	+	−	+
	VpJHY9	August 2017	+	−	−	+	+	+	+	+	+	+	+
	VpJHY10	August 2017	+	−	−	+	+	+	+	+	−	−	−
	VpJHY11	September 2017	+	−	−	+	+	+	+	+	−	−	−
	VpJHY12	September 2017	+	−	−	+	+	+	+	+	−	−	−
	VpJHY13	July 2018	+	−	−	+	+	+	+	+	−	−	−
	VpJHY14	July 2018	+	−	−	+	+	+	+	+	−	−	−
	VpJHY15	August 2018	+	−	+	+	+	+	+	+	+	+	+
	VpJHY16	August 2018	+	−	−	+	+	+	+	+	+	+	+
	VpJHY17	August 2018	+	−	−	+	+	+	+	+	−	−	−
	VpJHY18	September 2018	+	−	−	+	+	+	+	+	+	−	+
	VpJHY19	September 2018	+	−	−	+	+	+	+	+	−	−	−
	VpJHY20	September 2018	+	−	−	+	+	+	+	+	−	−	−
*Macrobrachium nipponense*	VpJHY21	July 2016	+	−	−	+	+	+	+	+	−	−	−
	VpJHY22	August 2016	+	−	−	+	+	+	+	+	−	−	−
	VpJHY23	August 2016	+	−	−	+	+	+	+	+	−	−	−
	VpJHY24	August 2017	+	−	−	+	+	+	+	+	−	−	−
	VpJHY25	September 2017	+	−	−	+	+	+	+	+	−	−	−
	VpJHY26	August 2018	+	−	−	+	+	+	+	+	−	−	+
	VpJHY27	August 2018	+	−	−	+	+	+	+	+	−	−	−
	VpJHY28	August 2018	+	−	−	+	+	+	+	+	−	−	−
	VpJHY29	August 2018	+	−	−	+	+	+	+	+	−	−	−
	VpJHY30	August 2018	+	−	−	+	+	+	+	+	+	−	+
	VpJHY31	September 2018	+	−	−	+	+	+	+	+	−	−	−
	VpJHY32	September 2018	+	−	−	+	+	+	+	+	−	−	−
*Penaeus vannamei*	VpJHY33	July 2016	+	−	−	+	+	+	+	+	−	−	−
	VpJHY34	August 2016	+	−	−	+	+	+	+	+	−	−	−
	VpJHY35	August 2016	+	−	−	+	+	+	+	+	+	+	+
	VpJHY36	August 2016	+	−	−	+	+	+	+	+	+	+	+
	VpJHY37	August 2016	+	−	−	+	+	+	+	+	−	−	−
	VpJHY38	June 2017	+	−	−	+	+	+	+	+	−	−	−
	VpJHY39	July 2017	+	−	−	+	+	+	+	+	+	+	+
	VpJHY40	July 2017	+		−	+	+	+	+	+	−	−	−
	VpJHY41	August 2017	+	−	−	+	+	+	+	+	+	+	+
	VpJHY42	August 2017	+	−	−	+	+	+	+	+	−	−	−
	VpJHY43	August 2017	+	−	−	+	+	+	+	+	−	−	−
	VpJHY44	August 2017	+	−	−	+	+	+	+	+	−	−	−
	VpJHY45	August 2017	+	−	−	+	+	+	+	+	−	−	−
	VpJHY46	June 2018	+	−	−	+	+	+	+	+	−	−	−
	VpJHY47	July 2018	+	−	−	+	+	+	+	+	−	−	−
	VpJHY48	August 2018	+	−	−	+	+	+	+	+	+	+	+
	VpJHY49	August 2018	+	−	−	+	+	+	+	+	−	−	−
	VpJHY50	August 2018	+	−	−	+	+	+	+	+	−	−	−
	VpJHY51	September 2018	+	−	−	+	+	+	+	+	−	−	+
*Macrobrachium rosenbergii*	VpJHY52	August 2016	+	−	−	+	+	+	+	+	−	−	+
	VpJHY53	September 2016	+	−	−	+	+	+	+	+	−	−	−
	VpJHY54	August 2017	+	−	−	+	+	+	+	+	−	−	−
	VpJHY55	July 2018	+	−	−	+	+	+	+	+	−	−	−
	VpJHY56	August 2018	+	−	−	+	+	+	+	+	+	−	+
	VpJHY57	August 2018	+	−	−	+	+	+	+	+	−	−	+
	VpJHY58	August 2018	+	−	−	+	+	+	+	+	−	−	−
	VpJHY59	September 2018	+	−	−	+	+	+	+	+	−	−	−

### Antimicrobial Susceptibility and Heavy Metal Tolerance

**FIGURE 2:**
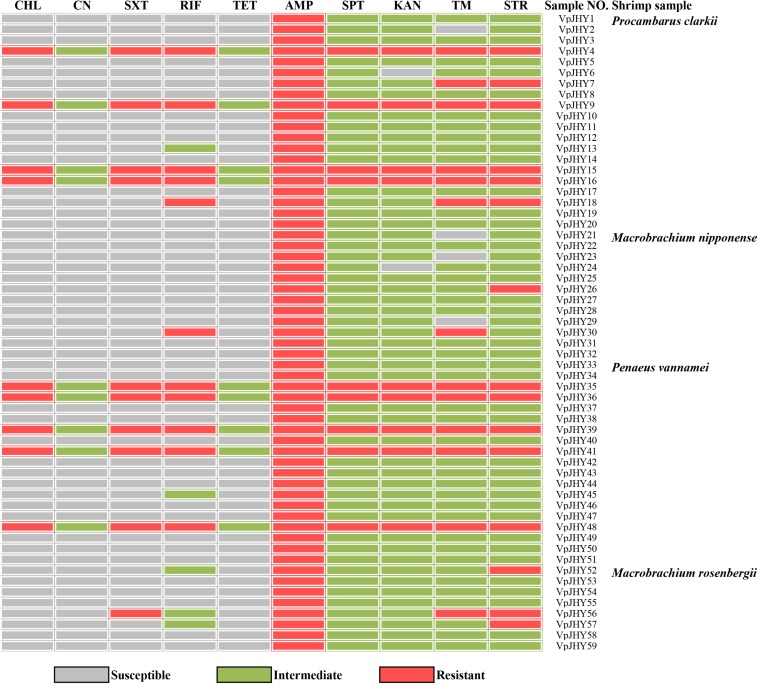
Drug resistance of *V. parahaemolyticus* isolates. CHL, chloramphenicol; CN, gentamicin; SXT, sulfamethoxazole-trimethoprim; RIF, rifampicin; TET, tetracycline; AMP, ampicillin; SPT, spectinomycin; KAN, kanamycin; TM, trimethoprim, and STR, streptomycin.

Our results revealed distinct antibiotic-resistance patterns for the 59 isolates that were positive for the highly conserved ICE genes. All isolates were AMP resistant, and resistance to STR, TM, RIF, and SXT was 25.4, 22.0, 18.6, and 16.9%, respectively; resistance to CHL, SPT, and KAN was 15.3% (Figure 2). We found that approximately 84.7, 81.3, 74.6, and 72.9% of the isolates exhibited intermediate susceptibility to SPT, KAN, STR, and TM, and while 15.3% of isolates showed intermediate patterns of susceptibility to CN and TET, none of the isolates was resistant to these two antibiotics. We found nine isolates tested positive for the three resistance genes *strA*/*strB*, *dfrA1*/*dfr18*, *sul2*, and these strains were resistant to CHL, SXT, RIF, AMP, SPT, KAN, TM, and STR, with intermediate susceptibility to CN and TET. BLAST analysis showed that the 16S rRNA gene sequence of isolate VpJHY15 shared 99% similarity with that from *Proteus vulgaris*^3^, and similarity for the other eight strains (VpJHY4, VpJHY9, VpJHY16, VpJHY35, VpJHY36, VpJHY39, VpJHY41, and VpJHY48) to *V. parahaemolyticus* was 99%.

Therefore, in this study, eight *V. parahaemolyticus* isolates tested positive for SXT/R391-like ICE conserved genes (*int*, *attR*, *traC*, *setR*, *traI*) and associated typical resistance genes (*strA*/*strB*, *dfrA1*/*dfr18*, *sul2*). These strains may be described as having MDR phenotypes because they were resistant to at least one agent in ≥3 categories of antimicrobial (Thapa Shrestha et al., 2015). These isolates were also examined for susceptibility to heavy metals (Malik and Aleem, 2011), we found minimal inhibitory concentrations (MICs) of 3200 μg/mL for Ni^2+^, Cr^3+^, Cd^2+^, Cu^2+^, Pb^2+^, and Mn^2+^; 1600 μg/ml for Zn^2+^; and 50 μg/ml for Hg^2+^ (Table 3). All strains were resistant to Zn^2+^ and Pb^2+^, and most also displayed tolerance to Cu^2+^ (87.5%), Cd^2+^ (75%), and Hg^2+^ (62.5%), while a few were resistant to Cr^3+^ (37.5%), Ni^2+^ (25%), and Mn^2+^ (12.5%). *V. parahaemolyticus* isolates derived from the four shrimp species showed tolerance to at least four heavy-metal agents (Table 4). While we found that resistance to heavy metals in *V. parahaemolyticus* did not vary with shrimp species, tolerance was shown to be very prevalent in ICE-positive strains with more than eight antibiotic resistance phenotypes. These contrasting results may be a result of inappropriate, variable releases of industrial wastes to aquaculture environments (Jiang et al., 2019), because industrial pollutants enhance selection for antibiotic resistance and *vice versa*. Abundant double-resistant bacteria threaten human health if contaminated products are consumed (Silva et al., 2018).

**TABLE 3:** Heavy metal resistance of *V. parahaemolyticus* isolates.

**Heavy metals**	**MIC (μg/mL)**										**Resistant**	
	**6.25**	**12.5**	**25**	**50**	**100**	**200**	**400**	**800**	**1600**	**3200**	***n***	**(%)**
Ni								a				
							1	5	1	1	2	25
Cr								a				
								5	1	2	3	37.5
Cd							a					
						1	1	4	2		6	75
Cu								a				
								1	2	5	7	87.5
Pb									a			
										8	8	100
Mn									a			
									7	1	1	12.5
Zn							a					
								7	1		8	100
Hg		a										
	2	1	3	2							5	62.5

*MIC, minimum inhibitory concentrations. ^*a*^Minimal inhibition concentration of standard strain *E. coli* K12.*

**TABLE 4:** Susceptibility of *V. parahaemolyticus* harboring the SXT/R391 family of integrative and conjugative elements to heavy metals and antibiotics.

**Sample**	**Heavy metal tolerance**	**Antimicrobial susceptibility**	**Source**
*Vibrio parahaemolyticus* JHY4	Cr, Cd, Cu, Pb, Zn, Hg	CHL, SXT, RIF, AMP, SPT, KAN, TM, STR	*Procambarus clarkii*
*Vibrio parahaemolyticus* JHY9	Ni, Cd, Cu, Pb, Zn	CHL, SXT, RIF, AMP, SPT, KAN, TM, STR	*Procambarus clarkii*
*Vibrio parahaemolyticus* JHY16	Cr, Cd, Cu, Pb, Zn, Hg	CHL, SXT, RIF, AMP, SPT, KAN, TM, STR	*Procambarus clarkii*
*Vibrio parahaemolyticus* JHY35	Cd, Cu, Pb, Zn	CHL, SXT, RIF, AMP, SPT, KAN, TM, STR	*Penaeus vannamei*
*Vibrio parahaemolyticus* JHY36	Cu, Pb, Mn, Zn, Hg	CHL, SXT, RIF, AMP, SPT, KAN, TM, STR	*Penaeus vannamei*
*Vibrio parahaemolyticus* JHY39	Cr, Cd, Pb, Zn	CHL, SXT, RIF, AMP, SPT, KAN, TM, STR	*Penaeus vannamei*
*Vibrio parahaemolyticus* JHY41	Cd, Cu, Pb, Zn, Hg	CHL, SXT, RIF, AMP, SPT, KAN, TM, STR	*Penaeus vannamei*
*Vibrio parahaemolyticus* JHY48	Ni, Cu, Pb, Zn, Hg	CHL, SXT, RIF, AMP, SPT, KAN, TM, STR	*Penaeus*

### Phylogenetic Relationships of Resistant *V. parahaemolyticus* Isolates

**FIGURE 3:**
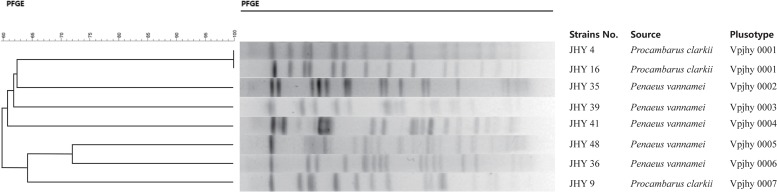
Phylogenetic tree showing ICEs-positive *V. parahaemolyticus* isolates.

Genomic DNA of the eight *V. parahaemolyticus* strains was individually digested with the restriction endonuclease *NotI*, and the resulting DNA fragments were resolved by PFGE. This analysis revealed different genomic finger prints of the strains tested (Figure 3). Fingerprinting analysis of the relatedness of the eight ICE-positive isolates produced 14 to 19 restriction bands that ranged from 20.5 to 1135 kb. Cluster analysis of the PFGE profiles revealed seven pulsotypes with ≥87% similarity, which indicates isolates belonging to the same epidemic strain (Seifert et al., 2005). The isolates were assigned to four distinct clusters, with 62.5% assigned to Clusters A to C and one that was more distantly related assigned to Cluster D. Simpson’s diversity index (0.9872) indicated high diversity among these isolates. Clustering analysis of the genomic fingerprints revealed eight distinguishable *NotI*-PFGE types, demonstrating that the MDR of ICEs-positive strains isolated for the prevalence analysis exhibited various genotypes. According to our previous studies, the presence or absence of ICEs has no significant relevance among these strains in terms of antimicrobial resistance (He et al., 2015a), it indicating that resistance determinants may spread among genetic lineages within the *V. parahaemolyticus* population. MGEs that carry resistance genes (Song et al., 2013) may be responsible for the high variation among the genotypes and resistance phenotypes of isolates. Therefore, we suggest future research to elucidate the precise mechanisms of resistance determinant transmission in *V. parahaemolyticus* populations.

## Conclusion

Bacteria secrete toxin proteins or effectors into external media or directly into eukaryotic target cells to facilitate adaption to environmental stress conditions; this response is key during the process of infection (Park et al., 2004; Cascales, 2008; Tseng et al., 2009). Previous studies have revealed that clinical isolates of *V. parahaemolyticus* produce beta-hemolysis in Wagatsuma agar, in a process known as the Kanagawa phenomenon that is linked to TDH-secreted proteins. These proteins have been recognized as primary virulence factors and effectors (Naim et al., 2001; Ono et al., 2006; Igbinosa and Okoh, 2008). Studies from different regions of the world show that *tdh* and/or *trh* genes are found in 90–99.8% of clinical strains, whereas only 0.2–10% of environmental *V. parahaemolyticus* isolates are potentially pathogenic, based on the presence of *tdh* and/or *trh* (Letchumanan et al., 2015b; Hu and Chen, 2016; Taiwo et al., 2017; Rortana et al., 2018; Jiang et al., 2019).

Previous studies disclosed that *V. parahaemolyticus* is a very diverse species and is an opportunistic pathogen in aquatic environments that is highly successful in adapting to changing environmental conditions (Song et al., 2013; Letchumanan et al., 2015a, b). Increasing aquaculture production and industrialization has led to large amounts of antibiotics being used to prevent or treat disease outbreaks in shrimp farming; consequently, multidrug resistant (MDR) and heavy metals resistant pathogens have emerged and posed serious problems in many aquatic systems. It revealed that bacteria could acquire resistance via conjugation or transformation, allowing them to adapt to the harsh environment and to cause a wide variety of diseases (Matyar, 2012). Incidents of human food poisoning from aquaculture products pose a becoming a serious clinical issue.

In the present study, we evaluated the prevalence, antimicrobial susceptibility, heavy metal resistance and genotypes of *V. parahaemolyticus* from four species of shrimp obtained from fish markets in Jiangsu (in 2016–2018), China. The results showed that ICEs-positive isolates have been given more antibiotic resistance, and the MDR strains also showed more heavy metal resistance. Consistent with previous reports (Song et al., 2013; Hu and Chen, 2016; Kang et al., 2018), we found that AMP resistance dominated among the isolates and was present in all samples tested. Although TET, sulfonamides, and quinolones are used widely in aquaculture (Holmström et al., 2003), we did not find evidence of resistance to TET in the *V. parahaemolyticus* isolates. Nine isolates that tested positive for all resistance genes (*strA*/*strB*, *dfrA1*/*dfr18*, *sul2*) exhibited intermediate susceptibility to CN and TET, indicating potential resistance to these two antibiotics. Our results for susceptibility of the *V. parahaemolyticus* isolates to CHL contrast with previous work (He et al., 2016), where we found all ICE-positive strains were resistant to CHL, possibly as a result of the ban, since 2002, of the use of CHL and derivatives (including chloramphenicol succinate) in the breeding industry in China (China Department of Agriculture, Bulletin No. 193). We suggest that ICE-positive *V. parahaemolyticus* isolates may indicate tolerance to heavy metal agents.

Wide usage of teracyclines, sulfonamides, and (fluoro) quinolones in aquaculture has been reported (Holmström et al., 2003). In addition, animal fecal used as fertilizer of aquaculture ponds is a common practice in integrated aquaculture-agriculture system herein. Manure and urine from field-herding cattles and goats were continuously discharged directly into ponds, which could change the bacterial community composition and bring more antimicrobials and even heavy metals in the aquaculture environment. In the *Vibrionaceae*, ICEs have been demonstrated to bestow resistance to multiple antibiotics and some complex new traits through horizontal gene transfer, which could be beneficial under certain environmental conditions (Makino et al., 2003; Balado et al., 2013). However, the present study revealed that ICEs are not the major transmission mediators of resistance to antibiotics or heavy metals. Thus, we speculate that many of the antibiotic resistance genes are intrinsic, and their expressions are activated when the environmental conditions became hostile. Furthermore, few of them are commonly transferred via conjugation or transformation. These data will aid future research in controlling of aquaculture diseases, forecasting food safety incidence and improving our understanding of *V. parahaemolyticus* prevalence and behavior in the aquaculture.

## Data Availability

The raw data supporting the conclusions of this manuscript will be made available by the authors, without undue reservation, to any qualified researcher.

## Author Contributions

YH, SW, XZ, and JZ participated in the design and/or discussion of the study. FS, BH, and XL carried out the major experiments. YH and SW analyzed the data. YH wrote the manuscript. SW, XZ, and JZ revised the manuscript. All authors read and approved the final version of the manuscript.

## Conflict of Interest Statement

The authors declare that the research was conducted in the absence of any commercial or financial relationships that could be construed as a potential conflict of interest.

## Footnotes

1 http://www.contigexpress.com

2 http://ncbi.nlm.nih.gov/BLAST

3 http://rdp.cme.msu.edu/
